# Editorial: At the borders of movement, art, and neurosciences, volume II

**DOI:** 10.3389/fpsyg.2026.1947462

**Published:** 2026-08-19

**Authors:** Guy Cheron, Jan De Maere, Bernard Andrieu, Carlos Cevallos, Ana-Maria Cebolla

**Affiliations:** 1 Laboratory of Movement Neurophysiology, Faculty of Human Movement Sciences, Université libre de Bruxelles, Brussels, Belgium; 2 Université Paris Cité, Paris, France

**Keywords:** creativity, emotion, movement, neuroaesthetics, perception

The relationship between science and art has long evolved through alternating periods of convergence and separation. At several key moments in history, artists and scientists have attempted to establish a common framework for understanding perception, creativity, movement, and human cognition. The Bauhaus movement provides one of the most influential examples of such a convergence. Through the work of artists such as Wassily Kandinsky, Paul Klee, and László Moholy-Nagy, artistic creation was closely associated with investigations into perception, geometry, color theory, and the underlying principles governing visual experience (Droste, 2002; Frank, 1984). During the same period, Salvador Dalí developed a profound interest in contemporary scientific discoveries, anticipating a future in which artistic intuition and scientific knowledge would mutually enrich one another (Ades, 1982).

These ambitions were abruptly interrupted by the political upheavals that marked the first half of the twentieth century. The closure of the Bauhaus in 1933 and the subsequent redirection of intellectual and financial resources toward military objectives illustrate how periods of conflict can profoundly alter the trajectory of scientific and cultural development (Droste, 2002).

Nearly a century later, the world appears to be entering another period of geopolitical instability. Across many nations, increasing investments in defense and security are becoming strategic priorities. While such developments may be justified by international circumstances, they also raise concerns regarding the balance of societal investments. Scientific research, healthcare, education, and artistic creation are among the fundamental drivers of human progress, fostering innovation, wellbeing, cultural identity, and social cohesion. The promotion of beauty, creativity, and knowledge should not be regarded as secondary objectives but rather as essential dimensions of human development.

In this context, the renewed dialogue between the arts and the sciences acquires particular significance. Recent advances in movement-cognitive neuroscience, neuroaesthetics, and the study of creativity have opened unprecedented opportunities to investigate the neural mechanisms underlying artistic perception, aesthetic experience, imagination, and creative action (Zeki et al., 2020; Chatterjee and Vartanian, 2014; Cebolla and Cheron, 2019; Cheron and De Maere, 2025). Beyond their respective domains, art and science share a common objective: the exploration of reality through complementary modes of inquiry. Their integration may therefore contribute not only to a deeper understanding of the human mind but also to the emergence of new educational, cultural, and societal perspectives capable of addressing the challenges of the twenty-first century. The recent launch of the Research Topic (RT) “*At the Borders of Movement, Art, and Neurosciences*” highlights the determination of the academic community to continue advancing knowledge despite the many constraints affecting contemporary research. Researchers are approaching the field of neuroaesthetics through a wide range of original and multidisciplinary perspectives, spanning both basic and applied science. The 12 articles published in the second volume of this Research Topic exemplify this growing momentum, placing the human brain and the recording of electroencephalographic (EEG) oscillations and gaze behavior at the center of investigations into creativity and the emergence of beauty.

The study of Nojo (a) proposes that aesthetic judgment emerges approximately 6–12 s after viewing onset and is not an instantaneous event but a dynamic process that eye-movement tracking can reveal. In a second study using eye tracking, Nojo (b) introduces the concept of Early Depth Engagement in Art Perception (EDEN) and investigates how engagement with pictorial depth unfolds over time during the viewing of artworks. A critical transition period around 6 s, was identified marking the shift from general exploration to structured, depth-centered viewing associated with higher aesthetic appreciation. Vaisvaser proposes that aesthetic experiences are not merely encounters with beauty or art, but fundamental embodied processes that promote psychological growth, self-integration, and therapeutic change. By combining neuroaesthetics, predictive processing, embodied cognition, and psychodynamic theory, the author argues that aesthetic experiences arise from the interplay between prediction, surprise, emotion, bodily sensations and meaning-making. The study of Nojo and Chan proposes and validates the RAIC hypothesis (Reading an Artist's Intention from the Composition): viewers experience a richer aesthetic response when their eye movements follow the compositional path intended by the artist. In other words, aesthetic appreciation is enhanced when the viewer's gaze is guided through the artwork in a manner consistent with expert understanding of its composition. This result corroborates the real impact of the connoisseurship experience (Cheron and De Maere, 2025). An additional point that may be particularly relevant to the interests of this Research Topic in neuroaesthetics is that the review by Arkhipova et al. suggests that many forms of artistic training (drawing, music composition, improvisation, creative writing) induce neural changes similar to those observed in highly creative individuals, supporting the idea that aesthetic and creative experiences can reshape large-scale brain networks through learning and practice such as the Default Mode Network (Vessel et al., 2012; Cela-Conde et al., 2013), associated with spontaneous idea generation, and the Executive Network (EN). EEG recordings, together with skin conductance, heart rate, and eye tracking, were used by Iosa et al. to investigate museum visitors viewing Egyptian mummies. Using EEG, they showed that viewing mummies increases visitors' cognitive engagement and emotional arousal, but do not induce strong negative emotional reactions such as disgust, irritation, or rejection. When the Eye-tracking identified the moment participants first looked at the exposed face, the skin conductance level, an index of autonomic emotional arousal, increased significantly and was associated with heightened cognitive effort (indexed by the presence of frontal theta EEG activity). A systematic review and meta-analysis of 928 patients with Parkinson's disease, Tang et al. (2026) evaluating the effects of art-based interventions (dance, music, theater, and multimodal artistic programs) on non-motor symptoms, highlighted a specific reduction in fear of falling but without significant effects on cognition and quality of life.

EEG analyses performed on elite table tennis athletes by Mao et al. revealed a significant reduction of the P3 component, a marker associated with attentional resource allocation. Under low cognitive load, athletes exhibited a clear subliminal priming effect, responding faster to congruent than incongruent subliminal stimuli. Under high cognitive load, this subliminal priming effect was markedly reduced or disappeared, indicating diminished unconscious processing.

The central thesis of the review of MacLellan et al. is that upper limb haptic interactions are not merely accessory to locomotion but constitute an important sensorimotor channel that contributes to adaptive locomotion, inter-individual coordination, anticipatory control, and rehabilitation. Human locomotion should therefore be viewed as a whole-body process integrating sensory information from both upper and lower limbs.

The perspective article of Gori and Santini argues that painting should not be viewed solely as a visual product but as the result of a complex interaction between multisensory perception, motor action, creativity, and technology. The authors emphasize that the act of painting involves continuous integration of visual, tactile, proprioceptive, and cognitive processes, transforming mental images into physical gestures on the canvas. They propose that the painter's movements, although invisible in the final artwork, are an essential component of artistic creation and perception.

In the systematic review and meta-analysis, Jia et al. showed that peripheral visual field loss (PFL) significantly impairs locomotion. Individuals with glaucoma or retinitis pigmentosa walk more slowly, experience more collisions with obstacles, and display altered gait patterns such as shorter strides, increased double-support time, and greater gait variability. These deficits become more pronounced in low-light, complex, or dual-task conditions. The authors conclude that peripheral vision is essential for safe mobility and fall prevention.

The study of Yang et al. proposes an AI-assisted design framework for modernizing the traditional Chinese Jiaoyi (folding chair) by combining Kansei Engineering, eye-tracking data, emotional preference analysis, and a CNN-GRU-Attention deep-learning model. The authors extracted emotional descriptors from web and social media data, identified three principal user preference dimensions: (1) flexible refinement, (2) uncompromising quality, and (3) ergonomic stability. They demonstrate how computational intelligence can support the preservation and modernization of cultural heritage products through data-driven emotional design.
